# The miR‐3127‐5p/p‐STAT3 axis up‐regulates PD‐L1 inducing chemoresistance in non‐small‐cell lung cancer

**DOI:** 10.1111/jcmm.13657

**Published:** 2018-05-04

**Authors:** Dongfang Tang, Dandan Zhao, Yun Wu, Ruyong Yao, Lin Zhou, Liming Lu, Wen Gao, Yifeng Sun

**Affiliations:** ^1^ Department of Thoracic Surgery Shanghai Key Laboratory of Clinical Geriatric Medicine HuaDong Hospital Affiliated with FuDan University Shanghai China; ^2^ Central Laboratory of Shanghai Chest Hospital Affiliated with Shanghai Jiaotong University Shanghai China; ^3^ Central laboratory of the Affiliated Hospital of Qingdao University Shanghai China; ^4^ Department of Thoracic Surgery Shanghai Chest Hospital Affiliated with Shanghai Jiaotong University Shanghai China

**Keywords:** immune escape, JAK/STAT3, lung cancer, microRNA‐3127‐5p, PD‐L1

## Abstract

It is less known about miRNA3127‐5p induced up‐regulation of PD‐L1, immune escape and drug resistance caused by increased PD‐L1 in lung cancer. In this study, lentivirus was transduced into lung cancer cells, and quantitative PCR and Western blot were used to detect the expression of PD‐L1. Then immunofluorescence assay was applied to detect autophagy, finally we explored the relationship between PD‐L1 expressions and chemoresistance in patients. As a result, we found that microRNA‐3127‐5p promotes pSTAT3 to induce the expression of PD‐L1; microRNA‐3127‐5p promotes STAT3 phosphorylation through suppressing autophagy, and autophagy could retaine pSTAT3 into the nucleus in miRNA‐3127‐5p knocked cells, and immune escape induced by elevated level of PD‐L1 results in chemoresistance of lung cancer. In conclusion, microRNA‐3127‐5p induces PD‐L1 elevation through regulating pSTAT3 expression. We also demonstrate that immune escape induced by PD‐L1 can be dismissed by corresponding monoclonal antibody.

## 1 INTRODUCTION

Lung cancer is a leading cause of tumour‐related mortality worldwide. Non‐small‐cell lung cancer (NSCLC) accounts for about 85% of all lung cancer cases.^1^ The epidermal growth factor receptor (EGFR) gene is one of the most common driver genes in NSCLC. Up to 47.9% of Asian NSCLC patients harbour EGFR mutation.^2^, ^3^ Fusion of the Echinoderm microtubule‐associated protein like‐4 (EML4) and anaplastic lymphoma kinase (ALK) represent another distinct mechanism of driver mutation in NSCLC, accounting for about 4%‐8.1% patients.^4^, ^5^ Although chemotherapy remains the main treatment of advanced NSCLC, small molecular tyrosine kinase inhibitors (TKIs) were recommended as the first‐line treatment of advanced NSCLC with druggable driver mutations. However, a majority of patients eventually develop acquired resistance and limited strategies are available to handle TKIs resistance. Novel and effective therapy for NSCLC is urgently warranted.

Although immune escape and increasement of PD1/PD‐L1 would be accompanied by drug resistance in lung cancer, it is more commonly induced in tumour and normal cells by cytokines, especially IFN‐γ.^6^ The complexity of PD‐L1 expression has made it difficult to identify the specific PD‐L1 expressing cells that contribute to escape from immune surveillance, which implies mechanistic and clinical importance for PD‐L1 expression may stratify response of patients to anti‐PD‐1/PD‐L1 immunotherapy.^7^ Past attempts to resolve this dilemma have been inconclusive. In addition, PD‐L1 on immune cells is expressed more frequently than that on tumour cells in patients with urothelial carcinoma and oesophageal squamous cell carcinoma, suggesting distinct extrinsic regulatory pathways are involved in tumour vs immune cell PD‐L1 induction.

Previously, we have demonstrated that overexpression of miR‐3127‐5p significantly reduced NSCLC cells proliferation, migration and motility in vitro and in vivo.^8^ In view of monoclonal antibody (mAb) blockade of programmed death1 (PD‐1) or its major ligand PD‐L1 can provoke durable antitumour responses in some cancer patients and tumour‐bearing mice.^9^, ^10^ In this study, we will illustrate the following issues: (i) The relationship between miR‐3127‐5p and PD‐L1; (ii) the specific mechanism of miR‐3127‐5p in regulating the expression of PD‐L1; (iii) the effectiveness of PD‐L1 monoclonal antibody on immune escape and drug resistance induced by PD‐L1 up‐regulation.

## 2 MATERIALS AND METHODS

### 2.1 Human samples

Human lung cancer and their corresponding non‐tumorous tissues were collected at the time of surgical resection from 64 patients with non‐small‐cell lung cancer from April 2016 to October 2017 at the Department of Thoracic Surgery of the HuaDong Hospital Affiliated to FuDan University. Human tissues were immediately frozen in liquid nitrogen and stored at −80°C refrigerator. Except the IA patients, others will receive postoperative treatment, lung adenocarcinoma will receive EGFR‐TKI or cisplatin‐based regimens according to EGFR mutation, lung squamous cell carcinoma will receive cisplatin‐based regimens. Generally, patients relapse within 3 months after the first course of chemotherapy is defined as chemoresistance; ≥3 months was sensitive. Signed informed consent was obtained from all patients and the study was approved by the Clinical Research Ethics Committee of FuDan University.

### 2.2 Cell culture and regents

Human non‐small‐cell lung cancer cell lines (A549, NCI‐H1299) cells were purchased from the Cell Resource Center, Shanghai Institute of Biochemistry and Cell Biology at the Chinese Academy of Sciences. Cells were maintained at 37°C in a humidified air atmosphere containing 5% carbon dioxide in RPMI1640 supplemented with 10% FBS. Rapamycin (R0395), and wortmannin (W3144) were purchased from Sigma.

### 2.3 RNA extraction and quantitative real‐time PCR

Total RNA was extracted from cultured cells using the Isolation Kit (Ambion; Life Technologies), and from formalin‐fixed and paraffin‐embed normal and human lung cancer specimens using the Recover All Total Nucleic Acid Isolation Kit (Ambion; Life Technologies). cDNA was synthesized from total RNA with specific stem‐loop primers and the TaqMan Reverse Transcription Kit (Applied Biosystems; Life Technologies).

The sequences of the primers were as follows:

PD‐L1, forward, 5′‐TGGCATTTGCTGAACGCATTT ‐3′;

Reverse, 5′‐ TGCAGCCAGGTCTAATTGTTTT ‐3′;

CD25, forward: 5′‐GTGGGGACTGCTCACGTTC‐3′,

Reverse: 5′‐CCCGCTTTTTATTCTGCGGAA‐3′;

GAPDH was used as an internal control and amplified with forward primer: 5′‐GGAGCGAGATCCCTCCAAAAT‐3′,

Reverse primer: 5′‐GGCTGTTGTCATACTTCTCATGG‐3′.

### 2.4 Deep sequencing

Genomic DNA and RNA was isolated using RNeasy Kit (Qiagen) according to manufacturer's instructions. RNA was reverse transcribed using Omniscript reverse transcriptase (Qiagen). Coding genomic sequences and cDNA of STAT3 were amplified and purified using the QIAquick PCR purification kit (Qiagen). Primer sequences are available upon request. Purified PCR products were sequenced with the ABI PRISM BigDye Terminator cycle ready reaction kit V3.1 (Applied Biosystems, Foster City, CA, USA) using the PCR primers as sequencing primers. The sequencing was performed on a 3130xl Applied Biosystems Genetic Analyzer, and the data were analysed with DNA Sequencing Analysis software, version 5.2 (Applied Biosystems) and Sequencher™ version 4.8 (Gene Codes Corporation, Ann Arbor, MI, USA).

### 2.5 Western blot analysis

According to standard Western blot procedures, briefly, proteins were separated by 8% SDS‐PAGE and then transferred to nitrocellulose membrane (Bio‐Rad). After blocking in 5% non‐fat milk, the membranes were incubated with the following primary antibodies: rabbit anti‐PD‐L1 monoclonal antibody (mAb; 1:100; Abnova), mouse anti‐GAPDH mAb (1: 10 000; Sigma). The proteins were visualized with enhanced chemiluminescence reagents (Pierce).

### 2.6 Virus packaging and infection

The fluorescent lentiviral vectors pLenti‐III‐mir‐blank, pLenti‐III‐mir‐3127‐5p and pLenti‐III‐mir‐3127‐5p‐off were purchased from Applied Biological Materials, Inc. (ABM, Canada). To produce lentivirus, all vectors were transduced into HEK‐293T cells with virus packaging plasmids using LentiFectin™ reagent. To generate stably transduced cell lines, the A549 and H1299 cells were infected with each lentivirus, and subjected to puromycin selection (A549: 0.8 mg/mL; H1299: 1.0 mg/mL) for 2 weeks.

### 2.7 Plasmid construction and luciferase assays

Luciferase reporter constructs containing the miR‐3127‐5p binding sites of miR‐3127‐5p mimics and negative control were synthesized by Gene Pharma (Shanghai, China). A549 and H1299 cells were co‐transduced with these reporter constructs and miR‐3127‐5p mimics or negative control using Lipofectamine 2000 (Invitrogen). At 48 hours after transfection, we examined luciferase activities using the Dual Luciferase Reporter Assay System (Promega) following the manufacturer's instructions. Each treatment was performed in triplicate, and activities were normalized to Renilla luciferase.

### 2.8 Flow cytometric analysis

Cells were collected by trypsinization and washed with ice‐cold PBS. The cells were then stained with PE‐labelled anti‐PD‐L1 mAb (29E. 2A3; BioLegend, San Diego, CA, USA), APC‐labelled anti‐PD‐1 mAb (EH12.2H7; BioLegend) or isotype control and analysed by flow cytometry. Labelled cells were analysed by a FACSCalibur flow cytometer (BD, San Jose, CA, USA) and FlowJo (Tree Star Inc., Oregon, and USA). Each assay was done in triplicate.

### 2.9 Immunofluorescence (IF)

Cells were fixed with 4% formaldehyde, permeabilized with 0.2% Triton and blocked with 2% BSA for the indicated times. Next, the prepared cells were stained with anti‐STAT3 (1:100), anti‐pSTAT3 (1:100), anti‐LC3 (1:100) at 4°C. The next day, the cells were incubated with FITC‐conjugated secondary antibody for 1 hour, and then observed under a fluorescence microscope. To label the nuclei, cells were counterstained with 4′, 6‐diamidino‐2‐phenylindole (DAPI, Invitrogen) and were visualized using a confocal microscope.

### 2.10 Microscopy

Cells were plated at low confluence in 12‐well plates (50 000 cells/well). On day2, cells were exposed to serum starvation (0% FBS), normal medium (10% FBS), or chloroquine (50 mmol/L) for 24 hours. Medium was removed, cells were washed with PBS and treated with 4% paraformaldehyde/PBS for 20 minutes at room temperature, washed and then permeabilized with 0.1% Triton X‐100 for 10 minutes. Cells were then blocked with 5% normal goatserum (Cell Signaling Technology) containing 0.3% Triton X‐100 in PBS for 60 minutes. Diluted primary antibody, antimouse LC3 A/B (Cell Signaling Technology), was applied in blocking buffer overnight at 4°C. Alexa Fluor‐555 secondary antibodies diluted in 1% normal goat serum in PBS were added for 1 hour at ambient temperature. Cells were fixed using Vectashield hard set mounting medium containing DAPI dye (Vector Laboratories). Images were acquired using confocal microscopy (Olympus FV‐1000) and overlaid using ImageJ.

### 2.11 Preparation of human peripheral blood T cells

Peripheral blood mononuclear cell (PBMC) was obtained by Ficoll density gradient centrifugation (1006.2 *g* × 30 min), and the cell concentration was adjusted to 2 × 10^6^/mL. Using the Easy Sep Human T Cell Enrichment Kit (STEMCELL, Inc., Canada) for T cell selection and isolation, obtaining human T cells with CD3^+^ T> 90%.

### 2.12 Cell viability assays

Cell viability reagent functions as a cell health indicator using the reducing power of living cells to quantitatively measure the proliferation of various human and animal cell lines. Anti‐human anti‐CD3 m Ab (0.5 μg/mL) was used to coated with 96‐well culture plate, 100 μL each well, overnight at 4°C, aspirated to the coating solution and washed twice with PBS. Peripheral blood T cells were adjusted to 1.2 × 10^5^/mL and seeded in antibody pre‐coated 96‐well cell culture plates (100 μL/well) for 3 days until T cells began to proliferate. And co‐cultured with miRNA‐3127‐5p transduced A549 cells according to the target ratio of A 549: T (1: 5). The anti‐PD‐L1 m Ab was designed to block PD‐1/PD‐L1 signal, the cells were cultured in 5% CO2 and incubated at 37°C for 3‐5 days. Then 10 μL of CCK8 reagents was added to every subset well, and continued to incubate for 4‐6 hours. The absorbance of the cells was quantitated in a microplate reader at 450 nm with a reference wavelength of 630 nm. Each subgroup has 3 holes.

### 2.13 Statistical analysis

Data were shown as mean ± SD unless otherwise noted, the Student's *t* test was used for statistical analysis, and all statistical analyses were performed with the SAS 9.4 software. *P* values were shown 2‐sided, statistical differences at *P* < .05 were considered to be significant. Graphical displays were prepared using Graph Pad Software (Graph Pad Software, Inc, La Jolla, CA, USA) to show the distributions of expression.

## 3 RESULTS

### 3.1 MicroRNA‐3127‐5p induces the up‐regulation of PD‐L1

**Figure 1:**
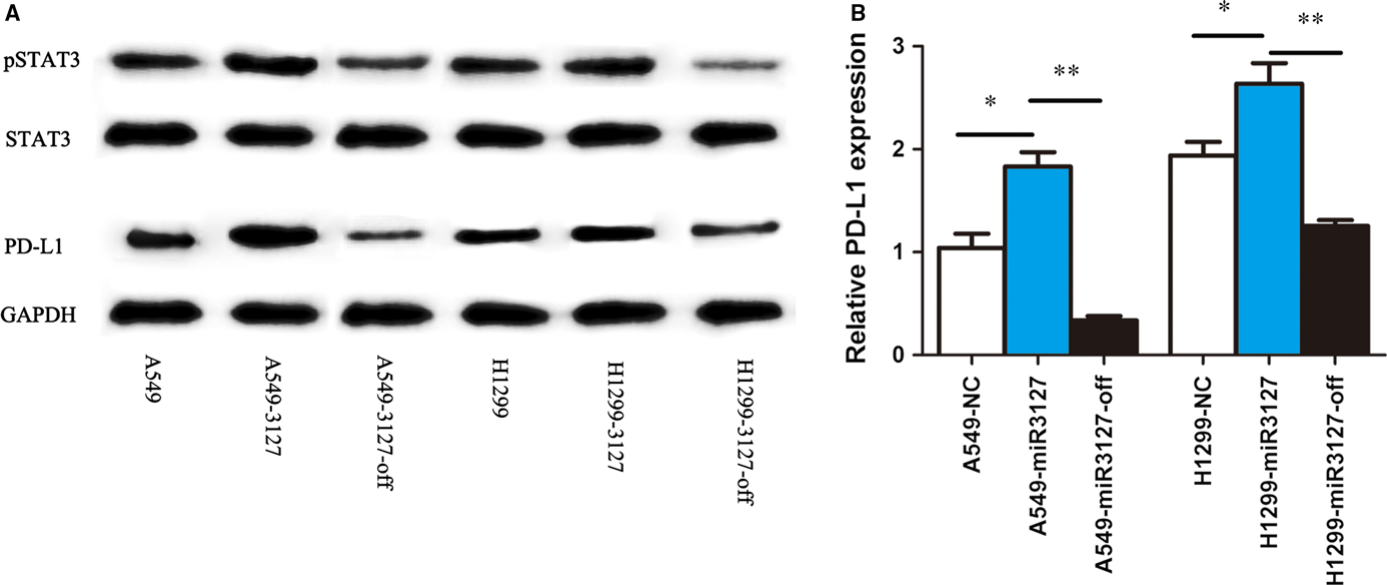
A, PD‐L1 increased significantly in exogenous miRNA‐3127‐5p transduced A549 and H1299 cells. In contrast, the expression of PD‐L1 was suppressed significantly when miRNA‐3127‐5p was knocked (*P* < .01); the expression of p‐STAT3 increased in miRNA‐3127‐5p transduced A549 and H1299 cells compared with knocked and empty vector control (*P* < .01), however, the expression of STAT3 did not change obviously. B, qPCR shows that PD‐L1 increased significantly in miRNA‐3127‐5p transduced A549 cells and H1299 cells (* represents *P* < .05; ** represents *P* < .001)

**Figure 2:**
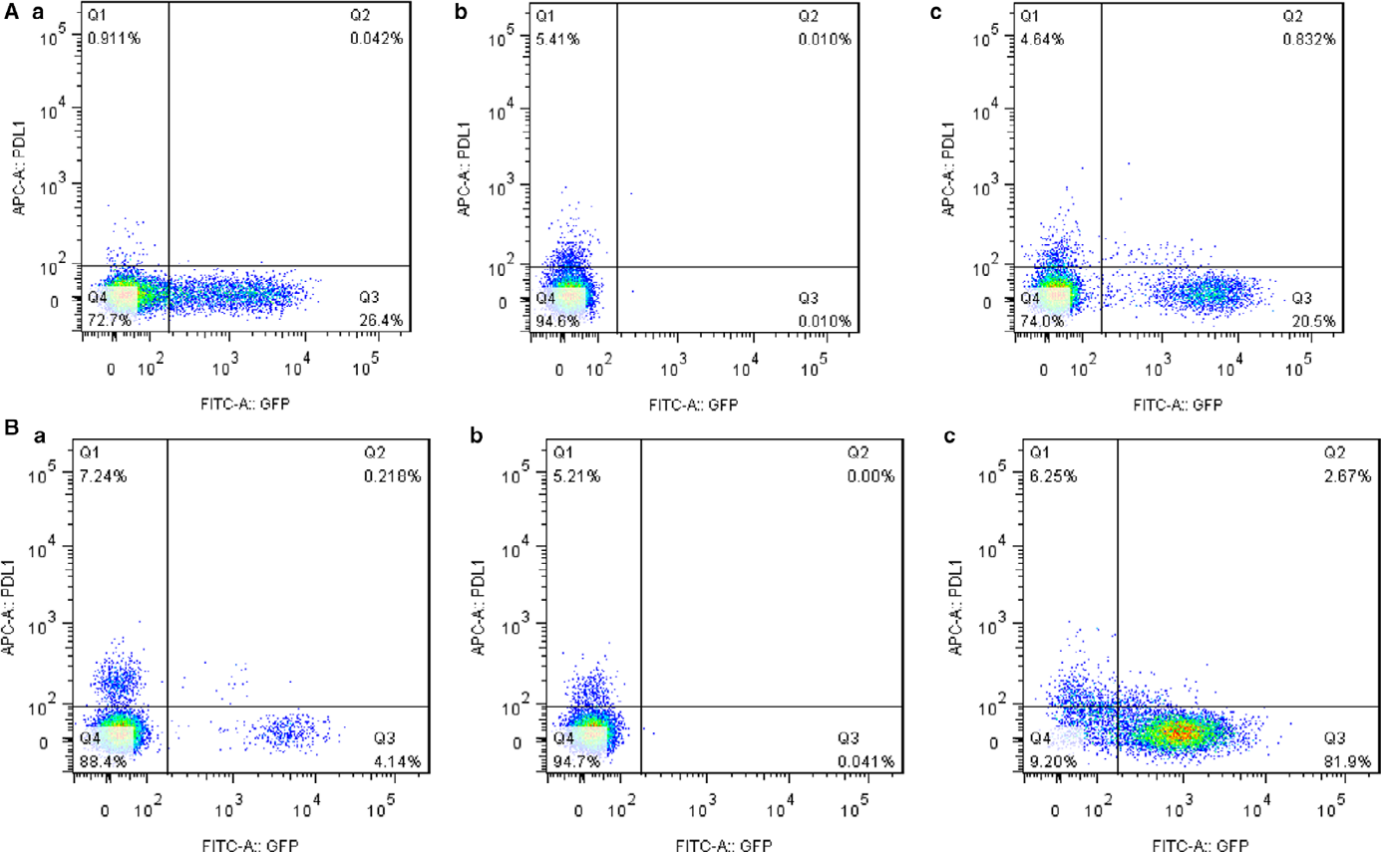
A, Flow cytometry shows that PD‐L1 induced by miRNA‐3127‐5p in A549 cells; a, PD‐L1 expression in miRNA‐3127‐5p‐knocked down A549 cells, b, PD‐L1 expression in A549 cells, c, PD‐L1 expression in miRNA‐3127‐5p transduced A549 cells. B, Flow cytometry shows that PD‐L1 induced by miRNA‐3127‐5p in H1299 cells; a, PD‐L1 expression in miRNA‐3127‐5p‐knocked down H1299 cells, b, PD‐L1 expression in H1299 cells, c, PD‐L1 expression in miRNA‐3127‐5p transduced H12999 cells

**Figure 3:**
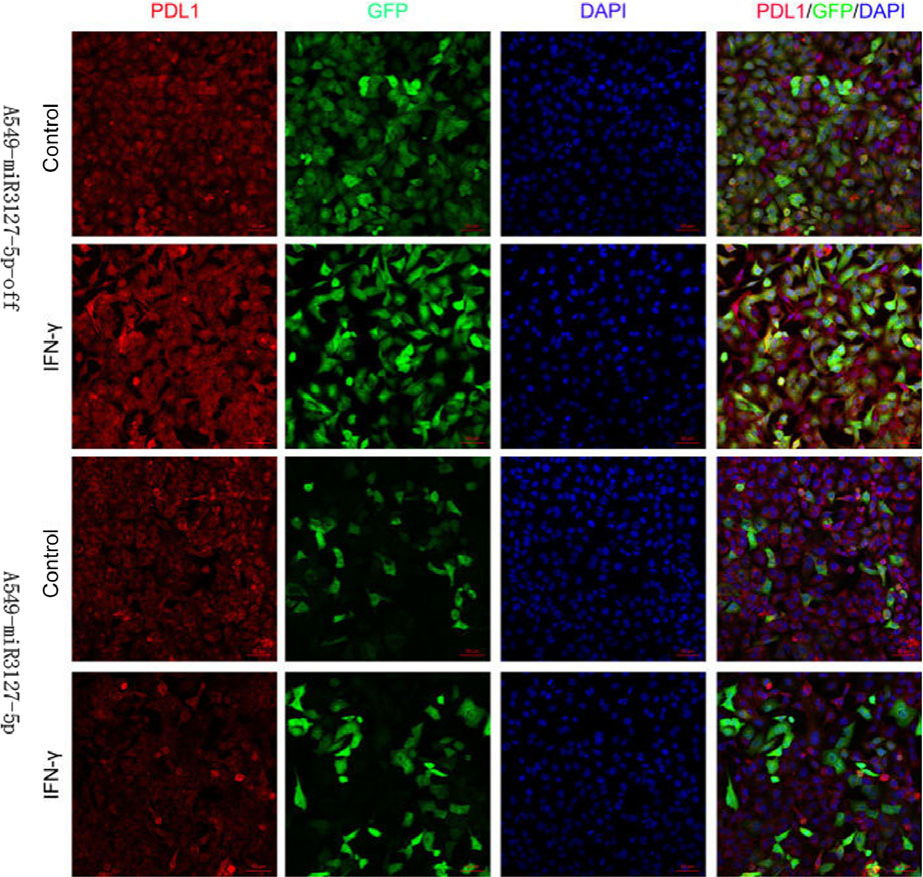
Immunofluorescence shows that miRNA‐3127‐5p induced more PD‐L1 presenting on the membrane of transduced A549 cells compared with knocked down; and INF‐γ as an exogenous stimulus, there is still no change after stimulation

MicroRNA‐3127‐5p‐lentiviruses were transduced in human NSCLC A549 and H1299 cells. We found that the expression of PD‐L1 was induced by exogenous miRNA‐3127‐5p in transduced A549 and H1299 cells. In contrast, the expression of PD‐L1 was significantly suppressed when miRNA‐3127‐5p was knocked (Figure 1A,B). Furthermore, the induction of PD‐L1 by miRNA‐3127‐5p was further confirmed by flow cytometry (Figure 2). Finally, we employed immunofluorescence to show the association between miRNA‐3127‐5p and PD‐L1 expression. Higher expression of PD‐L1 induced by miRNA‐3127‐5p was presented on the membrane of transduced A549 cells (Figure 3). Taken together, these results indicate that overexpression of miRNA‐3127‐5p may induce PD‐L1 expression.

### 3.2 MicroRNA‐3127‐5p promotes pSTAT3 to induce the expression of PD‐L1

**Figure 4:**
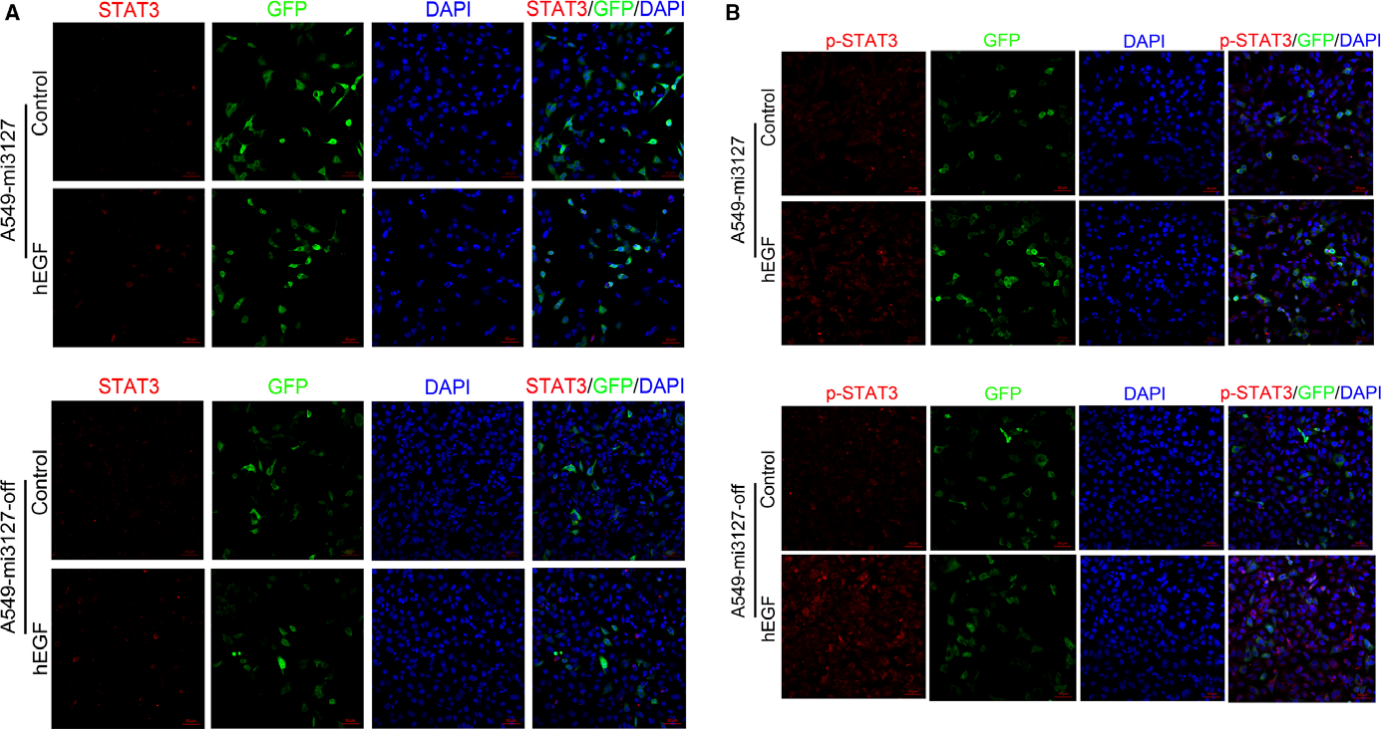
A, STAT3 was not significantly altered in the miRNA‐3127‐5p transduced A549 cells, and hEGF as an exogenous stimulus, 300 ng/mL for half an hour, there is still no change after hEGF stimulation. Immunofluorescence showed that STAT3 mainly distributed in the cytoplasm. B, Immunofluorescence showed that p‐STAT3 increased significantly in miRNA‐3127‐5p transduced A549 cells compared with knocked down and empty vector control. hEGF as an exogenous stimulus, 300 ng/mL for half an hour, the expression of pSTAT3 increased significantly after stimulation and mainly distributed in the nucleus

We next sought to explore the signalling pathways by which miRNA‐3127‐5p mediates expression of PD‐L1. To answer this question, we first accessed to literature. Previously, a constitutive oncogenic pathway has been reported to drive PD‐L1 expression through signal transducer and activator of transcription 3 (STAT3) signalling in lymphoma cells. In addition, STAT3 signalling is known to be a downstream target of miRNA‐3127 in lung cancer. We hypothesized that miRNA‐3127 regulates PD‐L1 expression by activating STAT3. Therefore, we detected the expression of STAT3 in miRNA‐3127‐5p transduced A549 and H1299 cells. As a result, we found that STAT3 was not significantly altered in the miRNA‐3127‐5p transduced lung cancer cells, and hEGF as an exogenous stimulus, 300 ng/mL for half an hour, there is still no change after hEGF stimulation. Immunofluorescence showed that STAT3 mainly distributed in the cytoplasm (Figure 4A). To further demonstrate the regulative mechanism, we examined the expression and distribution of p‐STAT3. As shown in Figure 1A,B the expression of p‐STAT3 increased in miRNA‐3127‐5p transduced lung cancer cells compared with knocked and empty vector control. hEGF as an exogenous stimulus, 300 ng/mL for half an hour, the expression of pSTAT3 increased significantly after stimulation. Immunofluorescence showed that pSTAT3 was significantly increased in the miRNA‐3127‐5p transduced cells and mainly distributed in the nucleus. Therefore, we speculated that microRNA‐3127‐5p promotes pSTAT3 to induce the expression of PD‐L1.

### 3.3 MicroRNA‐3127‐5p activates STAT3 phosphorylation through suppressing autophagy

**Figure 5:**
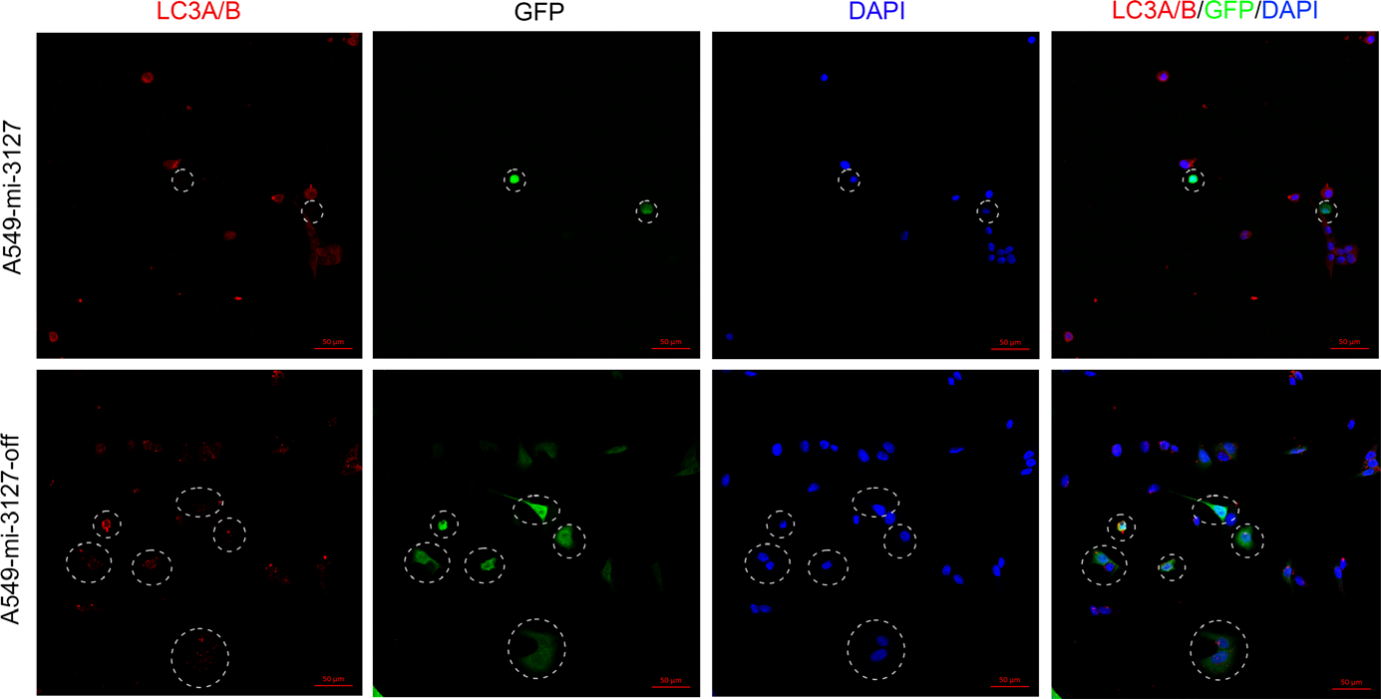
Autophagy weakened in miRNA‐3127‐5p transduced cells compared with knocked down

**Figure 6:**
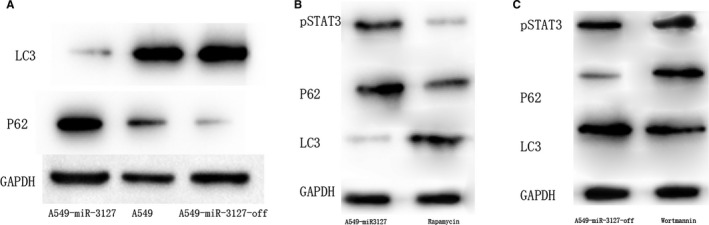
A, LC3 increased in miRNA‐3127‐5p knocked down cells, whereas, p62 decreased significantly in miRNA‐3127‐5p knocked down cells; B, when the miR‐3127 transduced cells were treated with autophagy activator rapamycin, the expression of LC3 increased and the expression of p62 and pSTAT3 decreased significantly (*P* < .001); C, When the miR‐3127 knock down cells were treated with the autophagy inhibitor wortmannin, the expression of LC3 decreased, the expression of p62 and pSTAT3 increased significantly (*P* < .001)

To explain this contradictory conclusion, we detect the autophagy phenomenon in miRNA‐3127‐5p transduced and knockout A549 cells. Serum starvation for 24 hours augmented autophagy in control cells as expected. As a result, we found that autophagy weakened in miRNA‐3127‐5p transduced cells compared with knocked down (Figure 5). To further validate the autophagic flux, we assessed the autophagy activity by measurement of the microtubule‐associated protein light chain 3 (LC3) as well as p62 protein level. As a result, LC3 increased in miRNA‐3127‐5p knocked down cells, whereas, the degradation protein of p62, an adaptor protein which serves as an autophagy receptor targeting ubiquitin proteins to autophagosomes for degradation; decreased significantly, indicating an enhanced autophagic flux (*P* < .001, Figure 6A).

To further examine whether activation of autophagy would suppress STAT3 phosphorylation or inhibiting of autophagy would activate STAT3 phosphorylation, we administered rapamycin which induces autophagy by inhibiting the mammalian target of rapamycin (mTOR) and Wortmannin which inhibits autophagy to cells. As a result, when the miR‐3127‐5p transduced cells were treated with autophagy activator rapamycin, the expression of LC3 increased and the expression of p62 and pSTAT3 decreased significantly (*P* < .001, Figure 6B). When the miR‐3127 knocked down cells were treated with the autophagy inhibitor wortmannin, the expression of LC3 decreased, the expression of p62 and pSTAT3 increased significantly (*P* < .001, Figure 6C).

Previously, frequent mutation of receptor protein tyrosine phosphatases (PTPRT) has been reported and provided a mechanism for STAT3 hyperactivation in head and neck cancer, so we also detected the mutation of PTPRT in human lung cancer cells, and did not find the same result as in head and neck cancer. Therefore, we elicited the conclusion that microRNA‐3127‐5p promotes STAT3 phosphorylation through suppressing autophagy, and autophagy could retaine pSTAT3 into the nucleus in miRNA‐3127‐5p knocked cells.

### 3.4 Up‐regulation of PD‐L1 induces immune escape resulting in lung cancer chemoresistance

**Figure 7:**
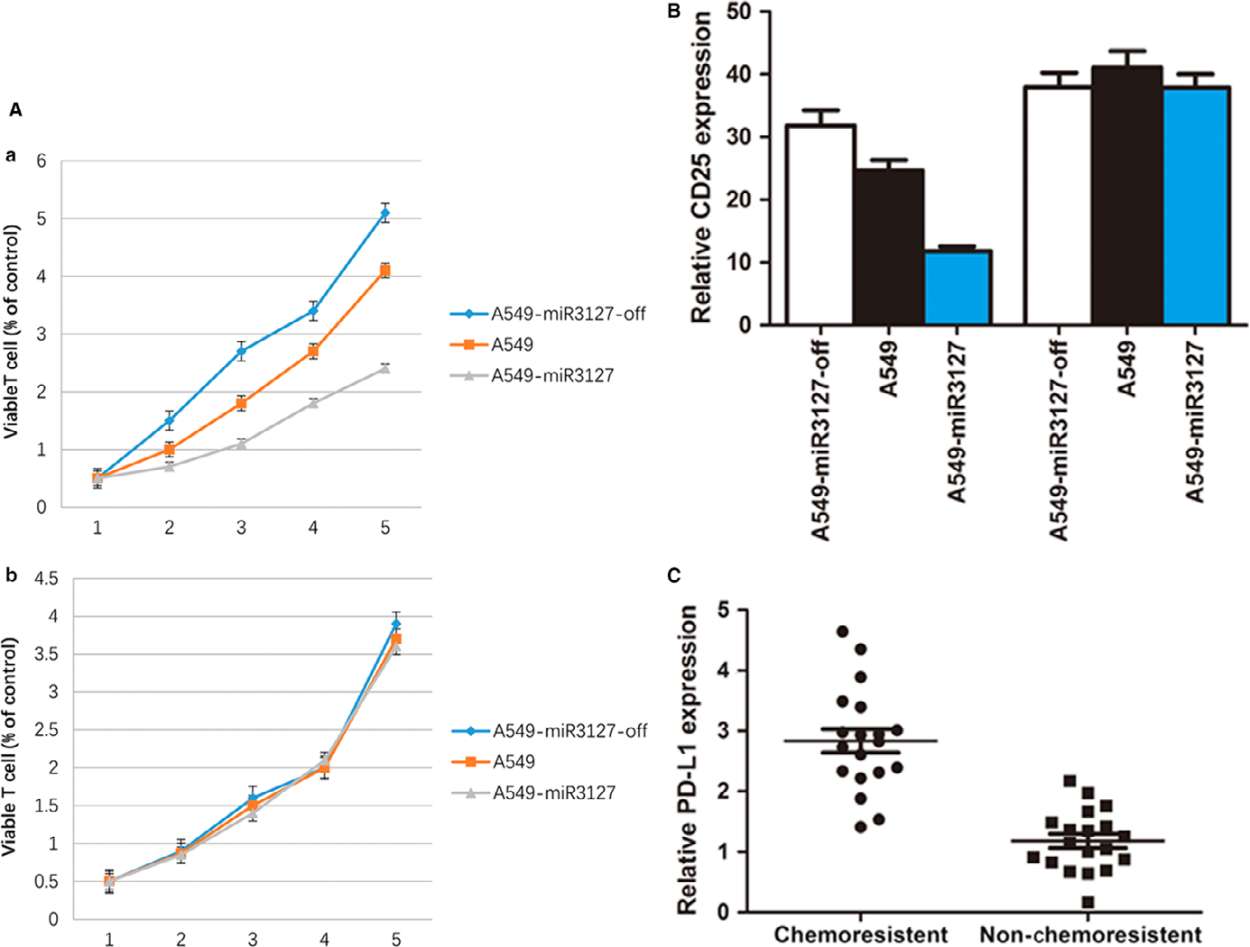
A‐a, PD‐L1 over‐expressing A549 cell line induced by miR3127‐5p inhibited T cell proliferation significantly; A‐b, the cell lines could relieve suppressive effect on CD3^+^ T cells after blocking by anti‐PD‐L1 mAb. B, the expression of CD25^+^ was significantly up‐regulated when PD‐L1 was blocked by monoclonal antibody in A549 cells (*P* < .001), A549 knock down cells (*P* < .05), A549 transduced cells (*P* < .001). C, PD‐L1 was up‐regulated significantly in patients who are chemoresistant (*P* < .001)

As PD‐L1 plays an important role in immune evasion, we further validated the immune escape function of PD‐L1 in lung cancer cells. We isolated and purified peripheral blood T cells, obtained human T cells CD3^+^ T> 90%. After 3 days of stimulation with stimulated anti‐CD3 mAb, CCK8 assay was performed and the results showed that T cells began to proliferate after activating; Then co‐cultured with miRNA‐3127‐5p transduced A549 cells, the training group was treated with anti‐PD‐L1 mAb to block the expression of PD‐L1 induced by miRNA‐3127‐5p. Thus we detected the cell proliferation and the expression of cell co‐stimulatory factor CD25^+^. As a result, PD‐L1 over‐expressing A549 cell line induced by miR3127‐5p inhibited T‐cell proliferation significantly (Figure 7A‐a), after blocking by anti‐PD‐L1 mAb; the cell lines could relieve suppressive effect on CD3^+^ T cells (Figure 7A‐b). And the expression of CD25^+^ was significantly up‐regulated when PD‐L1 was blocked by monoclonal antibody (Figure 7B).

**Figure 8:**
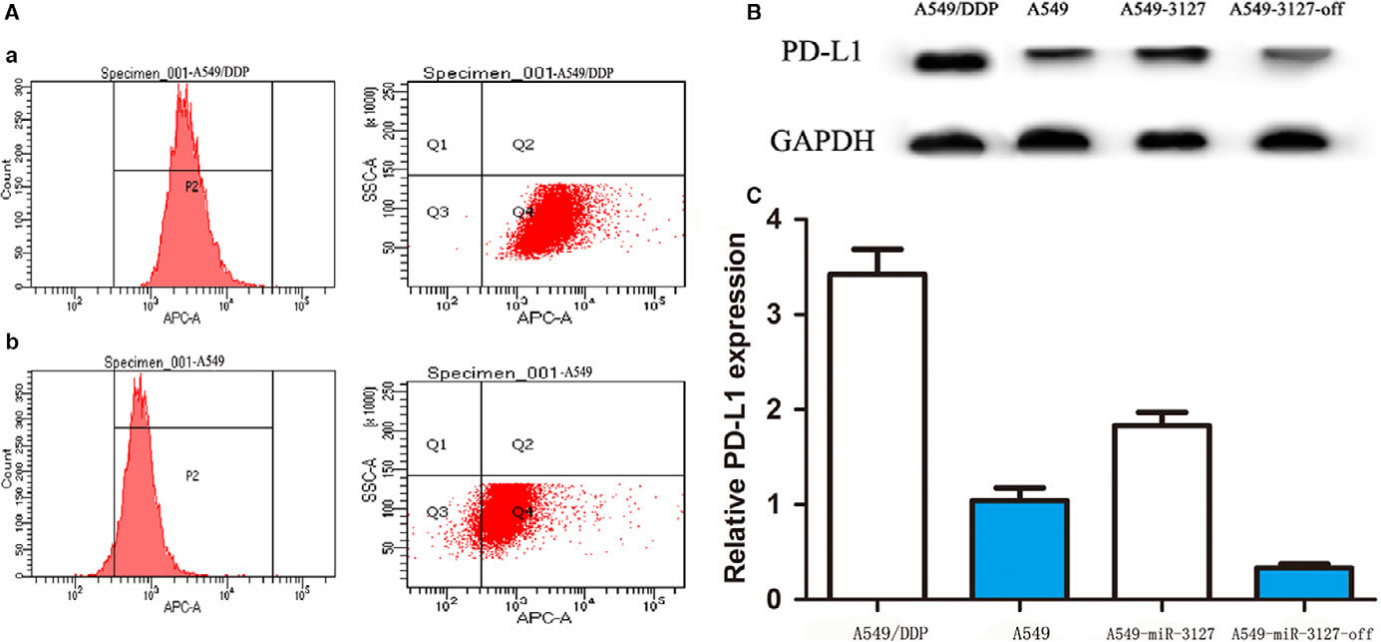
A, Flow cytometry shows that PD‐L1 in A549/DDP cells was higher than that of A549 cells; a: PD‐L1 expression in A549/DDP cells, b: PD‐L1 expression in A549 cells. B, Western blot shows that PD‐L1 expression in A549/DDP cells, A549 cells, A549‐miR‐3127‐5p transduced cells, A549‐miR‐3127‐5p knocked down cells. C, qPCR shows that PD‐L1 expression in A549/DDP cells, A549 cells, A549‐miR‐3127‐5p transduced cells, A549‐miR‐3127‐5p knocked down cells

We further examined the expression of PD‐L1 in lung cancer tissues and A549/DDP cells (Cisplatin‐resistant human lung cancer cells), and found that PD‐L1 was up‐regulated significantly in patients who are chemoresistant (*P* < .001, Figure 7C), the characteristics are shown in Table 1, and the expression of PD‐L1 in A549/DDP cells was higher than that of A549 cells and A549‐miR‐3127‐5p transduced cells, A549‐miR‐3127‐5p knocked down cells (*P* < .001, Figure 8A,B,C). Therefore, we reached the conclusion that up‐regulation of PD‐L1 induces immune escape resulting in lung cancer chemoresistance.

**Table 1:** The characteristics of patient

Variable	Patients (n)
Gender	
Male	36
Female	28
Age	
<64 y	30
≥64 y	34
Smoking	
Never	20
Ever	44
Histology	
Adenocarcinoma	37
Squamous cell carcinoma	27
Stage	
I	24
II	18
III	14
IV	8
EGFR mutation	
Y	12
N	52
Chemoresistance	
Y	25
N	39

## 4 DISCUSSION

In this study, we have demonstrated that microRNA‐3127‐5p promotes pSTAT3 to induce the expression of PD‐L1; microRNA‐3127‐5p could suppress autophagy and activate STAT3 phosphorylation. Finally, up‐regulation of PD‐L1 induces immune escape resulting in lung cancer chemoresistance and PD‐L1 monoclonal antibody could retrieve chemoresistance obviously.

Programmed cell death ligand‐1 (PD‐L1) has recently gained considerable attention for its role in tumour immune escape. Takahiro Ochiya has revealed that the miR‐197/CKS1B/STAT3 mediated network can drive tumour PD‐L1 expression^11^; in the further step, George Z. Rassidakis et al have demonstrated that PD‐L1 is commonly expressed and transcriptionally regulated by STAT3 and MYC in ALK‐negative anaplastic large‐cell lymphoma.^12^ Komohara et al also found that IL‐27/Stat3 axis induces expression of PD‐L1/2 on inFIltrating macrophages in lymphoma.^13^ And in domestic Huang et al demonstrated the EGFR pathway is involved in the regulation of PD‐L1 expression via the IL‐6/JAK/STAT3 signalling pathway in EGFR‐mutated non‐small cell lung cancer and oncogenic kinase NPM/ALK induction through STAT3 expression of immunosuppressive protein PD‐L1.^14^ Above all, we can summarize the conclusion as follows: (i) STAT3 pathway plays an important role in the regulation of PD‐L1 expression in lung cancer and lymphoma; (ii) PD‐L1 has significant inhibitory effect on tumour immunity. However, in this study we found that microRNA‐3127‐5p promotes pSTAT3 to induce the expression of PD‐L1 in lung cancer. The possible explanation is that pSTAT3 is the functional state of STAT3, which means that pSTAT3 directly plays the regulatory role. MicroRNA‐3127‐5p is involved in immune escape through JAK/STAT3 pathway indeed, which is not contradictory to the above conclusions.

Previously we reported that miRNA‐3127‐5p inhibits lung cancer cell invasion and proliferation which plays as the role of tumour‐suppressor gene^8^; then it is inferred that miRNA‐3127‐5p Should have inhibited PD‐L1 expression. However, the results showed that miRNA‐3127‐5p promoted the expression of PD‐L1. Therefore, we speculated that a variety of cytokines regulates the expression of PD‐L1; miRNA‐3127‐5p is only one of them.

Whereas how microRNA‐3127‐5p regulates the phosphorylation process of STAT3? Watanabe, et al found that Sesamin can induce autophagy in colon cancer cells by reducing tyrosine phosphorylation of EphA1 and EphB2;^15^ and Salomoni, et al found that mTORC1‐independent autophagy regulates receptor tyrosine kinase phosphorylation in colorectal cancer cells via an mTORC2‐mediated mechanism;^16^ Condorelli, et al also demonstrated that phosphorylation‐regulated degradation of the tumour‐suppressor form of PED by chaperone‐mediated autophagy in lung cancer cells.^17^ However, Jing, et al reported CLDN1 may increase drug resistance of non‐small‐cell lung cancer by activating autophagy via up‐regulation of ULK1 phosphorylation.^18^ On the basis of these reports, we found that: (i) phosphorylation and autophagy are closely related in various cancers; (ii) it is intended that decreasing autophagy induced and improved phosphorylation in colon cancer and lung cancer, whereas it is also reported that decreasing phosphorylation could induce autophagy. In this study, we found that the lower autophagy in lung cancer cells, the more obvious phosphorylation of STAT3; this coincides with previous reports, so we conclude that microRNA‐3127‐5p suppress autophagy activating STAT3 phosphorylation. However, autophagy is a part of cell self‐protection mechanism, whether autophagy induced phosphorylation; or the opposite, still needs further verification.

Graham et al demonstrated that activation of the PD‐1/PD‐L1 immune checkpoint confers tumour cell chemoresistance associated with increased metastasis;^19^ and Chen, et al also revealed that miR‐424(322) reverses chemoresistance via T cell immune response activation by blocking the PD‐L1 immune checkpoint.^20^ So we intended to prove that whether PD‐L1could lead chemoresistance in lung cancer and the effectiveness of PD‐L1 monoclonal antibody on immune escape and drug resistance induced by PD‐L1 increasing. As a result, we find that PD‐L1 monoclonal antibody could retrieve chemoresistance obviously.

Whether PD‐L1 antibodies can effectively against EGFR‐TKI resistance which caused by EGFR secondary mutations or it is possible to delay EGFR secondary mutations? Whether the combination of PD‐L1 with EGFR‐TKI has a more obvious effect on lung adenocarcinoma patients with EGFR mutations, it is our future direction of study in this field.

## CONFLICTS OF INTEREST

There is no conflict of interest for any of the Authors in any aspects of the article. Informed consent was obtained from all the patients. The ethical committee of our institute approved the research protocol for this study.
